# Supplementary material for: Estimating spatially disaggregated probability of severe COVID-19 and the impact of handwashing interventions: The case of Zimbabwe
Source: PLoS One. 2023 Nov 29;18(11):e0292644. doi: 10.1371/journal.pone.0292644 (PMC10686513; doi:10.1371/journal.pone.0292644)
Supplement: S1 Table — (DOCX) [file pone.0292644.s001.docx]

# Supporting Information

Table S1. Assumed hospitalization rates for symptomatic infections by age category. Estimates are based on Verity [(Verity *et al.*, 2020)](https://www.zotero.org/google-docs/?rRvQny) at and multiplied by 1.67 to reflect rates among symptomatic infections assuming 60% of infections are symptomatic.

Table S1. Assumed hospitalization rates for symptomatic infections by age category

| Age category | Hospitalization rate (%) |
| --- | --- |
| <20 | 0.3 |
| 20-24 | 0.8 |
| 25-29 | 1.7 |
| 30-34 | 2.7 |
| 35-39 | 3.8 |
| 40-44 | 4.8 |
| 45-49 | 6.5 |
| 50-54 | 9.7 |
| 55-59 | 12.0 |
| 60-64 | 17.0 |
| 65-69 | 19.5 |
| 70-74 | 24.3 |
| 75-79 | 29.5 |
| 80+ | 30.0 |
