# Supplementary material for: Estimating spatially disaggregated probability of severe COVID-19 and the impact of handwashing interventions: The case of Zimbabwe
Source: PLoS One. 2023 Nov 29;18(11):e0292644. doi: 10.1371/journal.pone.0292644 (PMC10686513; doi:10.1371/journal.pone.0292644)
Supplement: Attachment — Submitted filename: Comments by HT.pdf [file pone.0292644.s002.pdf]

Manuscript Number: PONE-D-22-34494

Full Title: Estimating spatially disaggregated probability of severe COVID-19 and the impact of handwashing interventions: The case of Zimbabwe

Short Title: The spatially disaggregated probability of severe COVID-19 and handwashing interventions in Zimbabwe

Corresponding Author: Sophie Ayling, Ph.D. Candidate

University College London

London, UNITED KINGDOM

Reviewer Comments:

In this article, the authors addressed to estimate of the potential impact of improved access to handwashing on the risk of respiratory illness and its knock-on impact on the risk of developing severe COVID-19 disease across Zimbabwe. A geospatial model that allows the estimation of differential clinical risk at the district level was considered, in which the ideas is sounding great. Thus, the authors need to consider the following minor concerns, and address them point by point prior they move for publication:

- The abstract needs to clearly address the specific spatial method suggested to achieve the proposed objectives.
- The contribution of the work is not clear as compared with the art of the works. For instance, it's better if the contribution of the latest work compared with the spatial-based work like (Mu X, Yeh AG-O, Zhang X (2021) and etc) is briefly explained.
- I suggest considering references that are more relevant to the current work related to spatial and cast out all which are not relevant.
- The discussion lacks references see the first paragraph of your discussion subsection.

- The datasets were taken from different sources, thus it’s better if the authors put support with the sources. The issue of the quality of the data needs to be well explained in a separate paragraph of the data source. Under the methodology the authors mentioned as they calculated the prevalence of risk factors and 158 comorbidities for severe COVID-19 at the district level, the steps how the calculation was made are not clearly addressed.
- The mathematical writing up of the entire formulas considered is not well written and clearly defining each and every term is also good. The methodology part clearly describing the models and formulas.
- Bring figures and describe them in the body of the manuscript before the references.
- The discussion part also needs to be well described as compared with the baselines. For instance, the first paragraph under the subsection should be compared with some state of the arts.
- Under the conclusion/discussion the authors mentioned as there is a relationship between access to handwashing facilities and respiratory illness, a 325 known risk factor for severe COVID-19.
- Justifying the existence of the spatial variation in the risk of severe COVID-19 needs to take into consideration.
- Better if the authors explain the spatial components taken into account in the latest work. For instance, the results, concluded as there are spatial differences in the risk of severe COVID-19. This needs to be verified through inferential spatial analysis.

Conclusion: I recommend the editor to consider the article for publication just after incorporating my major concerns.
