# Supplementary material for: Estimating spatially disaggregated probability of severe COVID-19 and the impact of handwashing interventions: The case of Zimbabwe
Source: PLoS One. 2023 Nov 29;18(11):e0292644. doi: 10.1371/journal.pone.0292644 (PMC10686513; doi:10.1371/journal.pone.0292644)
Supplement: Attachment — Submitted filename: Response to Reviewers Letter Form_160823.docx [file pone.0292644.s003.docx]

Dear Reviewers and Editorial Team,

Thank you for taking the time to read and review our manuscript “*Estimating spatially disaggregated probability of severe COVID-19 and the impact of handwashing interventions: The case of Zimbabwe”*.  We greatly appreciate the thoughtful, constructive, and detailed feedback. We have worked to address all of the comments received. Please find below the editor and reviewer comments with our responses.

Thank you for your consideration and we will be happy to address any additional comments or feedback.

## 1. Responses to Reviewer Comments

5. Review Comments to the Author

**Reviewer #1:** The paper discusses the severity of COVID-19 disease in different parts of Zimbabwe The disease varies substantially between individuals, with some are asymptomatic while others are fatal. Several risk factors have been identified that affect the progression of SARS-CoV-2 to severe COVID-19. To mention some age, smoking and presence of underlying comorbidities such as respiratory illness, HIV, anemia and obesity. Given that respiratory illness is one such comorbidity and is affected by hand hygiene, it suggests that improving access to handwashing could lower the risk of severe COVID-19 among a population. In the paper, the potential impact of improved access to handwashing on the risk of respiratory illness was estimated and handwashing knock-on impact on the risk of developing severe COVID-19 disease across Zimbabwe was estimated. they have used a geospatial model that allows to estimate differential clinical risk at the district level.

Results:- shows that the current risk of severe disease is heterogeneous across the country, due to differences in individual characteristics and household conditions. - demonstrates how household level improved access to handwashing could lead to reductions in the risk of severe COVID-19 of up to 16% from the estimated current levels across all districts. Taken alongside the likely impact on transmission of SARS-CoV-2 itself, as well as countless other pathogens,
this adds further support for the expansion of access to handwashing across the country.
It also highlights the spatial differences in risk of severe COVID-19, and concluded that it shows the opportunity for better planning to focus limited resources in high-risk areas in order to potentially reduce the number of severe cases.

my comments are:-
1)The DHS used is from 2015 while they could have used a recent one published on 2020.
2) The captions used for the figures are not well descriptive.

Thank you for this review.

1) In fact, DHS 2020 was still not yet available for Zimbabwe at the time of this study. Part of data collection seems to have been postponed due to the pandemic and the current update on DHS’ website is that it is ‘ongoing’ for 2022-23. Please see <https://dhsprogram.com/methodology/survey/survey-display-556.cfm> for reference.
2) Captions for the figures have been updated

**Reviewer #2**: Manuscript Number: PONE-D-22-34494

Reviewer Comments:

In this article, the authors addressed to estimate of the potential impact of improved access to handwashing on the risk of respiratory illness and its knock-on impact on the risk of developing severe COVID-19 disease across Zimbabwe. A geospatial model that allows the estimation of differential clinical risk at the district level was considered, in which the ideas is sounding great. Thus, the authors need to consider the following minor concerns, and address them point by point prior they move for publication:

• The abstract needs to clearly address the specific spatial method suggested to achieve the proposed objectives .

The abstract has been updated to describe the use Generalized Additive Models (GAMs) used to generate estimates of risk factors for severe COVID-19 at the district level.

• The contribution of the work is not clear as compared with the art of the works. For instance, it’s better if the contribution of the latest work compared with the spatial-based work like (Mu X, Yeh AG-O, Zhang X (2021) and etc) is briefly explained.

The contribution to the literature has been updated. Reference to Mu X, Yeh Ag-O, Zhang X(2021) has been added [4].

• I suggest considering references that are more relevant to the current work related to spatial and cast out all which are not relevant.

References have been updated throughout the document, we hope that the new reference list meets the reviewers’ satisfaction but please let us know if there are others you would like us to include.

• The discussion lacks references see the first paragraph of your discussion subsection.

Four references have been added to the first paragraph of the discussion section. Two have been added with relation to the statement that COVID-19 severity is highly variable between individuals, and two have been added with regards to GAM.

• The datasets were taken from different sources, thus it’s better if the authors put support with the sources. The issue of the quality of the data needs to be well explained in a separate paragraph of the data source. Under the methodology the authors mentioned as they calculated the prevalence of risk factors and 158 comorbidities for severe COVID-19 at the district level, the steps how the calculation was made are not clearly addressed.

Thank you for this comment. Table 1 has been added to the body of the manuscript in the data section which outlines the data description, types and source of each of the data sources used for ease of reference. Information on quality of the datasets is accessible on the source websites which are publicly available. We have also expanded the summary of the methodological apporach at the start of the methods section, and added a paragraph in the limitations section on the limitations of the data itself that was used.

Regarding the ‘158 comorbidities’ – apologies if this was not clear, there are not 158 comorbidities in the model. In line 58, you can note that we draw attention to “hypertension, diabetes, cardiovascular disease and chronic respiratory disease” as comorbidities that exist. However, the data for which we have comorbidities that are used in the model is on HIV prevalence (without Viral Load Suppression), anaemia prevalence, obesity prevalence and respiratory illness prevalence (section beginning “comorbidities and factors for the probability of severe COVID-19) i.e. 4 comorbidities in total. We hope this is now clearer in the text but let us know if this needs further clarification

• The mathematical writing up of the entire formulas considered is not well written and clearly defining each and every term is also good. The methodology part clearly describing the models and formulas.

The mathematical formulae have been simplified for clarity in lines 225 and 234

• Bring figures and describe them in the body of the manuscript before the references.

Figures have been moved and described in the body of the manuscript accordingly.

• The discussion part also needs to be well described as compared with the baselines. For instance, the first paragraph under the subsection should be compared with some state of the arts.

Several references have been added to the discussion section on other studies which look at spatial disparities in the severity of COVID 19 in Spain, India and the US. In addition some clinical meta analyses studies are also added which seek to identify the clinical risk factors associated with higher severity in symptoms.

• Justifying the existence of the spatial variation in the risk of severe COVID-19 needs to take into consideration.

It is well understood that there is spatial variation in the distribution of risk factors for COVID-19 and every other disease. As such, it is highly likely that there will be spatial variation in risk of severe COVID-19. This has also been referenced with studies from other country contexts in the discussion section. (India, Spain and the US).

• Better if the authors explain the spatial components taken into account in the latest work. For instance, the results, concluded as there are spatial differences in the risk of severe COVID-19. This needs to be verified through inferential spatial analysis.

We have now added more detail to the methods section, including further details of the datasets used.

Conclusion: I recommend the editor to consider the article for publication just after incorporating my major concerns.

Thank you

## 1. Editorial comments and responses

Additional Editor Comments:

Dear Ms. Sophie Ayling,

Thank you for submitting your manuscript to PLOS ONE.

I have completed my evaluation of your manuscript. The reviewers recommend reconsideration of your manuscript following carful revision. I invite you to resubmit your manuscript after addressing the comments below. Please resubmit your revised manuscript by March 15, 2023.

Dealing with the estimation of spatially disaggregated probability of COVID-19 infection is an important research topic. Here the authors report the impact of improved access to handwashing on the risk of respiratory illness with spatial focus on COVID-19.

1) Abstract
- Please split this into introduction, methods, results and conclusions

Thank you, the format of the abstract has been adjusted accordingly

2) Introduction
- This is a very good background, but the following should be addressed
Update the figures in the first paragraph using recent number Thank you, addressed
ii) some paragraphs lack references, such as the fourth paragraph Thank you, addressed
iii) clearly state the research problem The research problem being addressed is the need to understand the likely spatially disaggregated impacts of SARS-CoV-2 in terms of disease severity in a population. A sentence has been added to this effect in the second paragraph of the introduction.

3) Data and Methods
- Move the “Ethics Statement” at the end of this Section, i.e. before the Result Section Thank you, addressed
- Line #131, don’t you think DHS2015 is an outdated data? Thank you, this comment has been addressed in the earlier response to reviewers. 1)	In fact, DHS 2020 was still not yet available for Zimbabwe at the time of this study. Part of data collection seems to have been postponed due to the pandemic and the current update on DHS’ website is that it is ‘ongoing’ for 2022-23. Please see https://dhsprogram.com/methodology/survey/survey-display-556.cfm for reference.
- Line #147, how the intentionally displaced DHS cluster location taken into account in the interpolation process? Unfortunately, there is not much that can be done about this, as displacement is an intrinsic part of the DHS methodology in order to preserve respondent privacy, and hence it has been mentioned in the study limitations. Please see report 7 from the DHS <https://dhsprogram.com/pubs/pdf/SAR7/SAR7.pdf> . However, the same report states that *“Analysis of both simulated and real DHS household survey data shows that the GPS coordinate displacement process produces data with displaced distances that are uniformly distributed. Furthermore, the addition of 1% of rural points that are displaced up to 10 km—for purposes of reducing disclosure risk in rural areas—affects very few points and does not change the overall average distribution of rural coordinates. Analysis of the effect of adding restrictions to the displacement process to prevent points from being displaced across administrative boundaries shows that, in most cases, adding restrictions does not change the average displacement, as long as the units used for the viii restrictions are not too small.”* References have also been added to other literature showing that the use of interpolated DHS clusters in this way is accepted practice.
- Line #154, the provided Github link not working. Apologies, the link has now been updated.
- Line #158, are your sample representative to perform district level estimation? The use of Generalized Additive modeling for DHS data at lower sub-national units is now well established in the Spatial modeling literature. For example Takahashi et al (2016) used DHS and GAM to quantify spatial patterns of measles vaccinations in ten contiguous countries in the Africa Great lakes region at subnational administrative units; Yankson et al also used the 2016 DHS in Ghana and binomial logistic regression for mapping malaria prevalence at high resolution; in the DRC, Utazi et al used DHS data to estimate the coverage of the third dose of diphtheria-tetanus-pertussis containing vaccine (DTP3) at 1x1km spatial resolution using a Gaussian process.
- Line #178-180, you state about the spatial model, but unable to its clear description. How the model stated/formulated? Spatial modeling was conducted on the DHS data to generate continuous estimates of risk factors which were then aggregated to district level. In all cases, the spatial modeling involved generalized additive models which included a bivariate smooth on latitude and longitude as implemented elsewhere. We hope that the revised manuscript, which now includes an extended summary of the analysis done, improves clarity.

- Line #222, the counterfactual scenarios are similar across the country. Since there is a considerable variation in handwashing practice in Zimbabwe (see your statement line #233-244), how your assumed scenarios will be valued?

While it is true that the baseline levels of handwashing are variable across the country, for the purposes of the counterfactual analysis, rather than assuming that handwashing could be improved everywhere by some absolute amount, we model scenarios where handwashing is improved to a given level throughout the country. We believe this is a more realistic approach as in reality interventions are likely to be focused in underperforming areas. Leading to larger improvements in those areas relative to better performing areas.

4) Results
- Fig2 (top-left), write the correct label for x- and y-axis Latitude and longitude have been written out in full as axis labels.
- Line #242. Handwashing risk highly linked with household wealth. How the authors justify the non-significant link between poverty and respiratory illness?

The poverty covariate data used in the modeling were only available at the ward level which masks variation between DHS clusters. It is therefore plausible that any association at the household or cluster level could also be masked.

5) Discussion
- Lacking to compare/citation with other studies, i.e., line #301 Citations have now been added
- Why no limitations discussion here? Please add a Limitation section. Limitations were previously described as part of the discussion section, they have now been separated into their own section.


[Note: HTML markup is below. Please do not edit.]

## 2. Journal requirements

1. Please ensure that your manuscript meets PLOS ONE's style requirements, including those for file naming. The PLOS ONE style templates can be found at

<https://journals.plos.org/plosone/s/file?id=wjVg/PLOSOne_formatting_sample_main_body.pdf> and

[https://journals.plos.org/plosone/s/file?id=ba62/PLOSOne_formatting_sample_title_authors_affiliations.pdf](https://nam11.safelinks.protection.outlook.com/?url=https%3A%2F%2Fjournals.plos.org%2Fplosone%2Fs%2Ffile%3Fid%3Dba62%2FPLOSOne_formatting_sample_title_authors_affiliations.pdf&data=05%7C01%7Csayling%40worldbank.org%7C387e84753e024d40092408db0df0488d%7C31a2fec0266b4c67b56e2796d8f59c36%7C0%7C0%7C638119098180047318%7CUnknown%7CTWFpbGZsb3d8eyJWIjoiMC4wLjAwMDAiLCJQIjoiV2luMzIiLCJBTiI6Ik1haWwiLCJXVCI6Mn0%3D%7C3000%7C%7C%7C&sdata=jAbA%2FEIAj6hIppwryKXPNKRo2yLRaDHFQNxFrbmHIIU%3D&reserved=0)

2. Please provide additional details regarding participant consent. In the ethics statement in the Methods and online submission information, please ensure that you have specified (1) whether consent was informed and (2) what type you obtained (for instance, written or verbal, and if verbal, how it was documented and witnessed). If your study included minors, state whether you obtained consent from parents or guardians. If the need for consent was waived by the ethics committee, please include this information.

All the data sources used in this analysis were secondary. Therefore all participant consent will have been gained according to DHS data collection procedure. Please see <https://dhsprogram.com/Methodology/Protecting-the-Privacy-of-DHS-Survey-Respondents.cfm>

If you are reporting a retrospective study of medical records or archived samples, please ensure that you have discussed whether all data were fully anonymized before you accessed them and/or whether the IRB or ethics committee waived the requirement for informed consent. If patients provided informed written consent to have data from their medical records used in research, please include this information.

Not applicable

3. We note that the grant information you provided in the ‘Funding Information’ and ‘Financial Disclosure’ sections do not match.

When you resubmit, please ensure that you provide the correct grant numbers for the awards you received for your study in the ‘Funding Information’ section.

Noted

4. Thank you for stating the following financial disclosure:

"We would like to acknowledge the support of the Global Water Security and Sanitation Partnership (GWSP) of the World Bank and the ieConnect for Impact Program  funded with UK aid from the UK government in completing this work. "

Please state what role the funders took in the study.  If the funders had no role, please state: "The funders had no role in study design, data collection and analysis, decision to publish, or preparation of the manuscript."

If this statement is not correct you must amend it as needed.

Please include this amended Role of Funder statement in your cover letter; we will change the online submission form on your behalf.

The funders had no role in study design, data collection and analysis, decision to publish, or preparation of the manuscript

5. Thank you for stating the following in your Competing Interests section:

"NO authors have competing interests"

Please complete your Competing Interests on the online submission form to state any Competing Interests. If you have no competing interests, please state "The authors have declared that no competing interests exist.", as detailed online in our guide for authors at <http://journals.plos.org/plosone/s/submit-now>

 This information should be included in your cover letter; we will change the online submission form on your behalf.

This statement has been included in the revised cover letter.

6. In your Data Availability statement, you have not specified where the minimal data set underlying the results described in your manuscript can be found. PLOS defines a study's minimal data set as the underlying data used to reach the conclusions drawn in the manuscript and any additional data required to replicate the reported study findings in their entirety. All PLOS journals require that the minimal data set be made fully available. For more information about our data policy, please see <http://journals.plos.org/plosone/s/data-availability>.

Upon re-submitting your revised manuscript, please upload your study’s minimal underlying data set as either Supporting Information files or to a stable, public repository and include the relevant URLs, DOIs, or accession numbers within your revised cover letter. For a list of acceptable repositories, please see <http://journals.plos.org/plosone/s/data-availability#loc-recommended-repositories>. Any potentially identifying patient information must be fully anonymized.

Important: If there are ethical or legal restrictions to sharing your data publicly, please explain these restrictions in detail. Please see our guidelines for more information on what we consider unacceptable restrictions to publicly sharing data: <http://journals.plos.org/plosone/s/data-availability#loc-unacceptable-data-access-restrictions>. Note that it is not acceptable for the authors to be the sole named individuals responsible for ensuring data access.

We will update your Data Availability statement to reflect the information you provide in your cover letter.

All data used in this study is publicly available and is available from the weblinks provided in Table 1 that has been updated accordingly since the last submission. The GitHub link provided within the paper also provides the code for replicability.

7. We note that Figures 1, 3, 4, 5 and 6 in your submission contain [map/satellite] images which may be copyrighted. All PLOS content is published under the Creative Commons Attribution License (CC BY 4.0), which means that the manuscript, images, and Supporting Information files will be freely available online, and any third party is permitted to access, download, copy, distribute, and use these materials in any way, even commercially, with proper attribution. For these reasons, we cannot publish previously copyrighted maps or satellite images created using proprietary data, such as Google software (Google Maps, Street View, and Earth). For more information, see our copyright guidelines: <http://journals.plos.org/plosone/s/licenses-and-copyright>.

We require you to either (1) present written permission from the copyright holder to publish these figures specifically under the CC BY 4.0 license, or (2) remove the figures from your submission:

    1. You may seek permission from the original copyright holder of Figures 1, 3, 4, 5 and 6 to publish the content specifically under the CC BY 4.0 license.

 We recommend that you contact the original copyright holder with the Content Permission Form (<http://journals.plos.org/plosone/s/file?id=7c09/content-permission-form.pdf>) and the following text:

“I request permission for the open-access journal PLOS ONE to publish XXX under the Creative Commons Attribution License (CCAL) CC BY 4.0 (<http://creativecommons.org/licenses/by/4.0/>). Please be aware that this license allows unrestricted use and distribution, even commercially, by third parties. Please reply and provide explicit written permission to publish XXX under a CC BY license and complete the attached form.”

Please upload the completed Content Permission Form or other proof of granted permissions as an "Other" file with your submission”

In the figure caption of the copyrighted figure, please include the following text: “Reprinted from [ref] under a CC BY license, with permission from [name of publisher], original copyright [original copyright year].”

    2. If you are unable to obtain permission from the original copyright holder to publish these figures under the CC BY 4.0 license or if the copyright holder’s requirements are incompatible with the CC BY 4.0 license, please either i) remove the figure or ii) supply a replacement figure that complies with the CC BY 4.0 license. Please check copyright information on all replacement figures and update the figure caption with source information. If applicable, please specify in the figure caption text when a figure is similar but not identical to the original image and is therefore for illustrative purposes only.

The following resources for replacing copyrighted map figures may be helpful:

USGS National Map Viewer (public domain): [http://viewer.nationalmap.gov/viewer/](https://nam11.safelinks.protection.outlook.com/?url=http%3A%2F%2Fviewer.nationalmap.gov%2Fviewer%2F&data=05%7C01%7Csayling%40worldbank.org%7C387e84753e024d40092408db0df0488d%7C31a2fec0266b4c67b56e2796d8f59c36%7C0%7C0%7C638119098180047318%7CUnknown%7CTWFpbGZsb3d8eyJWIjoiMC4wLjAwMDAiLCJQIjoiV2luMzIiLCJBTiI6Ik1haWwiLCJXVCI6Mn0%3D%7C3000%7C%7C%7C&sdata=rdgmkQ4iAIrgQ%2FkCSXP0gHpQFXSAZKE%2FZx%2FOZw1h4qM%3D&reserved=0)

The Gateway to Astronaut Photography of Earth (public domain):  [http://eol.jsc.nasa.gov/sseop/clickmap/](https://nam11.safelinks.protection.outlook.com/?url=http%3A%2F%2Feol.jsc.nasa.gov%2Fsseop%2Fclickmap%2F&data=05%7C01%7Csayling%40worldbank.org%7C387e84753e024d40092408db0df0488d%7C31a2fec0266b4c67b56e2796d8f59c36%7C0%7C0%7C638119098180047318%7CUnknown%7CTWFpbGZsb3d8eyJWIjoiMC4wLjAwMDAiLCJQIjoiV2luMzIiLCJBTiI6Ik1haWwiLCJXVCI6Mn0%3D%7C3000%7C%7C%7C&sdata=dhFJNcO5wKoTY7aeEfq0Cz1DhdpD9zCMt9vFQQPkXb8%3D&reserved=0)

Maps at the CIA (public domain): [https://www.cia.gov/library/publications/the-world-factbook/index.html](https://nam11.safelinks.protection.outlook.com/?url=https%3A%2F%2Fwww.cia.gov%2Flibrary%2Fpublications%2Fthe-world-factbook%2Findex.html&data=05%7C01%7Csayling%40worldbank.org%7C387e84753e024d40092408db0df0488d%7C31a2fec0266b4c67b56e2796d8f59c36%7C0%7C0%7C638119098180047318%7CUnknown%7CTWFpbGZsb3d8eyJWIjoiMC4wLjAwMDAiLCJQIjoiV2luMzIiLCJBTiI6Ik1haWwiLCJXVCI6Mn0%3D%7C3000%7C%7C%7C&sdata=nna8iCKvPGttD1kO6ojcg3yMh1Dajsv5vtSZE0J7vV8%3D&reserved=0)  and [https://www.cia.gov/library/publications/cia-maps-publications/index.html](https://nam11.safelinks.protection.outlook.com/?url=https%3A%2F%2Fwww.cia.gov%2Flibrary%2Fpublications%2Fcia-maps-publications%2Findex.html&data=05%7C01%7Csayling%40worldbank.org%7C387e84753e024d40092408db0df0488d%7C31a2fec0266b4c67b56e2796d8f59c36%7C0%7C0%7C638119098180203545%7CUnknown%7CTWFpbGZsb3d8eyJWIjoiMC4wLjAwMDAiLCJQIjoiV2luMzIiLCJBTiI6Ik1haWwiLCJXVCI6Mn0%3D%7C3000%7C%7C%7C&sdata=aVattKWeHs3YqMnm11Zgjfd3M2MjdKFjdcJx6qBZS4c%3D&reserved=0)

NASA Earth Observatory (public domain): <http://earthobservatory.nasa.gov/>

Landsat: [http://landsat.visibleearth.nasa.gov/](https://nam11.safelinks.protection.outlook.com/?url=http%3A%2F%2Flandsat.visibleearth.nasa.gov%2F&data=05%7C01%7Csayling%40worldbank.org%7C387e84753e024d40092408db0df0488d%7C31a2fec0266b4c67b56e2796d8f59c36%7C0%7C0%7C638119098180203545%7CUnknown%7CTWFpbGZsb3d8eyJWIjoiMC4wLjAwMDAiLCJQIjoiV2luMzIiLCJBTiI6Ik1haWwiLCJXVCI6Mn0%3D%7C3000%7C%7C%7C&sdata=p8f1pv9%2Fbe5fvbxLrmqcZpAaus77GwD3SJkEXq0%2Flbs%3D&reserved=0)

USGS EROS (Earth Resources Observatory and Science (EROS) Center) (public domain): [http://eros.usgs.gov/#](https://nam11.safelinks.protection.outlook.com/?url=http%3A%2F%2Feros.usgs.gov%2F%23&data=05%7C01%7Csayling%40worldbank.org%7C387e84753e024d40092408db0df0488d%7C31a2fec0266b4c67b56e2796d8f59c36%7C0%7C0%7C638119098180203545%7CUnknown%7CTWFpbGZsb3d8eyJWIjoiMC4wLjAwMDAiLCJQIjoiV2luMzIiLCJBTiI6Ik1haWwiLCJXVCI6Mn0%3D%7C3000%7C%7C%7C&sdata=H%2BftkIlKyrOVEh4n9iHyjZ8eJ2sU7yplDuV0B7%2Bs7sk%3D&reserved=0)

Natural Earth (public domain): [http://www.naturalearthdata.com/](https://nam11.safelinks.protection.outlook.com/?url=http%3A%2F%2Fwww.naturalearthdata.com%2F&data=05%7C01%7Csayling%40worldbank.org%7C387e84753e024d40092408db0df0488d%7C31a2fec0266b4c67b56e2796d8f59c36%7C0%7C0%7C638119098180203545%7CUnknown%7CTWFpbGZsb3d8eyJWIjoiMC4wLjAwMDAiLCJQIjoiV2luMzIiLCJBTiI6Ik1haWwiLCJXVCI6Mn0%3D%7C3000%7C%7C%7C&sdata=DaXFV1WE%2FYBuXKP25SAd2pJoZc2IcYZ0oro2N620ClI%3D&reserved=0)

Relevant Basemap attribution has been added.

8. Please review your reference list to ensure that it is complete and correct. If you have cited papers that have been retracted, please include the rationale for doing so in the manuscript text, or remove these references and replace them with relevant current references. Any changes to the reference list should be mentioned in the rebuttal letter that accompanies your revised manuscript. If you need to cite a retracted article, indicate the article’s retracted status in the References list and also include a citation and full reference for the retraction notice.

References have now been checked and updated accordingly, thank you.
